# Deciduous afforestation as a natural climate solution: impacts on biomass and carbon sequestration in boreal forests of Canada

**DOI:** 10.1186/s13021-025-00385-2

**Published:** 2026-01-03

**Authors:** Francois du Toit, Nicholas C. Coops, Christopher Mulverhill, Aoife Toomey

**Affiliations:** 1 Department of Forest Resources Management, Faculty of Forestry, University of British Columbia, 2424 Main Mall, Vancouver, BC V6T 1Z4 Canada; 2 BP, 501 Westlake Park Boulevard, Houston, TX 77079-2696 USA

**Keywords:** Natural climate solutions (NCSs), 3-PG, Physiological growth model, Afforestation

## Abstract

**Background:**

Rising temperatures and altered precipitation patterns are expected to have profound impacts on the composition and condition of boreal forests. As a result there are growing needs for climate adaptation strategies in boreal forest management; one potential solution to achieve these goals is the utilization of nature-based climate-informed adaption solutions including afforestation using deciduous species which can help offset carbon emissions and sequester carbon at an increased rate. Deciduous afforestation has the potential to allow mangers to adapt fire-risk, while increasing carbon storage. Here, we investigated the impact of deciduous compared to coniferous afforestation on biomass accumulation in the Canadian boreal using a process-based model (3-PG). 3-PG utilises physiological principals to predict the growth of individual species across a variety of climate scenarios. This approach is valuable for projecting forest growth under changing climate, as it can account for plant responses to environmental factors which may not be captured by empirical models based on historical data. We simulated forest growth under three future climate scenarios to 2080, and compared the aboveground biomass (AGB, tons of Dry Matter per hectare; tDM ha^−1^) accumulated to baseline estimates using locally adapted coniferous species. In addition, we investigated the modelled effects of converting from conifer to deciduous species on stand level soil water and vapor pressure deficit responses to climate.

**Results:**

We found that deciduous simulations sequester more carbon under all climate scenarios, with the greatest difference occurring in the warmest scenario (171 tDM ha^−1^ for coniferous species compared to 347.1 tDM ha^−1^ for deciduous species). Coniferous species were generally more water stressed than deciduous species; conifers were generally 65.6% more stressed compared to deciduous species in August under the warmest climate scenario, while northern sites were less stressed than southern sites.

**Conclusions:**

Simulations such as these highlight the importance of modelling and consideration of different planting scenarios in decision-making to ensure successful resource allocation. They also demonstrate the potential of nature-based adaptation solutions projects, and the role deciduous afforestation can play in provision of habitat, modifying wildfire risk and northern boreal biomass and timber supply.

**Supplementary Information:**

The online version contains supplementary material available at 10.1186/s13021-025-00385-2.

## Background

The Canadian boreal forest, spanning roughly 280 Mha across northern latitudes represents a significant portion of the worlds forest area [11, 49]. While these forests are adapted to cold climates, nutrient-poor soils, and frequent wildfire, they face major threats from climate change [12, 49]. Projected increases in winter temperatures of up to 8 °C and altered precipitation patterns may push key species beyond their climate envelope, negatively impacting photosynthesis, growth, and survival [73, 75]. Recent studies have shown that under warmer, drier conditions, historically dominant boreal species such as balsam fir, white spruce, paper birth, and eastern white pine lose substantial biomass share compared to species such as oak (*Quercus*) and maple (*Acer*) which increase in abundance [75].

In addition to increasing temperatures, forest fires are likely to increase in terms of occurrence, area burned, and severity over the next few decades [73]. The increase in frequency and severity of fires can cause changes in forest succession and species composition. Species such as aspen (*Populus tremuloides* Michx.) and birch (*Betula pendula* Roth) may outcompete conifers when hot, dry conditions prevent establishment of seedlings [74], especially if the interval between stand-replacing fires becomes too short to permit sexual maturation of trees [73]. Research has shown that deciduous trees can dominate postfire seedling communities in black spruce stands following high severity fires, which reduce the depth of organic layers in soil and therefore stored carbon on the landscape [46, 79]. In contrast, moist sites with low fire severity were more likely to return to pre-fire composition [47]. The increase of fire severity coupled with the direct effects of climate warming and drying has the potential to amplify the effects of shifting forest trajectories towards increased ‘deciduousness’, which can persist for decades [2].

Given the projected changes in climate and wildfire frequency, there is a growing need for climate adaptation strategies in boreal forest management. A potential solution to achieve these goals is the utilization of natural climate solutions (NCS, [26, 35]). NCS are defined as conservation, restoration, and improved land management practices (also known as pathways, [35]). These pathways are applicable in forests, grasslands, agricultural areas, and wetlands, and provide additional climate mitigation beyond business as usual [26]. Forest options offer two-thirds of cost-effective NCS mitigation needed to hold warming to below 2 °C [26, 35], and include both improved forest management (such as adaptive species management), and restoration of forest cover through both afforestation and reforestation.

Afforestation is an example of a nature-based adaptation strategy and is the process of establishing forest in areas that have not been historically forested, while reforestation takes place in areas that were historically forested, but are not currently forest [90]. Currently, several initiatives in Canada, such as the federal 2 billion trees program, British Columbias’s Forest Carbon Initiative, and Ontario’s 50 million Tree program are aimed at fulfilling international commitments and increasing carbon stocks in the forest, and align with the implementation of NCS in Canada [80]. Given that climate change has the potential to change forest succession and ecosystem dynamics, efforts related to promoting forest regeneration and carbon sequestration should consider the long-term effects of changing climate and disturbance regimes [6].

Deciduous tree cover is likely to increase in North American forests over time [63, 75]; this shift may enhance forest resilience and increase sequestration potential. Deciduous trees may reduce the risk of fire compared to mature conifers through the increasing of leaf moisture, lowering flammability as well as reducing the overall fuel load [7, 10]. Fires within deciduous-dominated stands can be smaller, and lower-intensity, which move more slowly compare to coniferous dominated stands [38]. Additionally, studies suggest that converting forests from coniferous to deciduous species can have a significant effect on the proportion of burned areas per year, converting forest composition at a rate of 0.1–0.4% per year may be sufficient to maintain constant burn rates across the boreal [7, 34]. Finally, the shift from slow-growing black spruce to fast-growing deciduous species can result in a net increase of carbon storage by a factor of five over the disturbance cycle [58].

Building on the positive implications associated with planting deciduous species, as well as Canada’s commitment to reforestation and afforestation, it is valuable to investigate the potential of afforestation using deciduous species as a nature-based adaptation solution that contributes to climate change mitigation. While the primary objective of this study is to evaluate the carbon storage potential of afforestation with deciduous species in the Canadian boreal, carbon storage can also be used as a metric for assessing the effectiveness of reforestation efforts, thereby indicating whether associated co-benefits such as enhanced forest resilience, improved habitat quality, and increased protection against wildfire can be realized. Deciduous afforestation can play a role as a nature-based adaptation solution that contributes both carbon sequestration and to long-term ecosystem stability under future climate conditions.

To effectively predict the long-term viability and carbon sequestration potential of different afforestation strategies under future climate, it is crucial to understand the physiological responses of tree species to changing environmental conditions, particularly water availability. Soil water content and Vapor Pressure Deficit (VPD) are key climatic variables that directly influence tree growth, water stress, and mortality, which are major determinants of the overall carbon balance in forests [84, 92]. Therefore, examining the spatial and temporal patterns of these modifiers to growth can provide critical insights into the resilience and performance of coniferous versus deciduous species, informing robust species selection for afforestation and reforestation strategies. Furthermore, mixed forests incorporating both coniferous and deciduous planting can be investigated, as mixed forests could provide an additional opportunity to future-proof our forests.

Du Toit et al. [27] furthered the methodology developed by Drever et al. [26] to provide an assessment of afforestation as a natural climate solution in the Canadian boreal. This research specifically extends that work by providing a comparative analysis of deciduous versus coniferous afforestation strategies, investigating the impacts of species conversion on stand-level aboveground biomass accumulation and critical physiological responses (soil water and vapor pressure deficit) under varying future climate scenarios. We use a process-based model (3-PG), which utilises physiological principals to predict the growth of individual species across a variety of climate scenarios, including outside their current native range [54, 55, 87]. Simulations are conducted for the establishment of deciduous stands, mixed deciduous-coniferous stands, and a baseline of locally adapted coniferous stands. We evaluate carbon sequestration and soil water dynamics under three future climate scenarios to 2080, and compare the aboveground biomass (AGB, reported as tons of dry matter, tDM) accumulated under each simulation. Our focus is the impact on above ground biomass; however we recognise that afforestation of deciduous stands will also impact albedo as well as below ground carbon and dead organic matter neither of which are the focus here. A key benefit of our approach is that it enables the comparison of future afforestation strategies with baseline conditions, providing insight into the potential of planting deciduous species for increased carbon sequestration, as well as adaptation to a changing climate. Additionally, we examine the modelled effects of species conversion on stand-level soil water availability and vapor pressure deficit responses, which are important factors given projected changes in precipitation patterns and frequency.

## Methods

### Study area

**Fig. 1:**
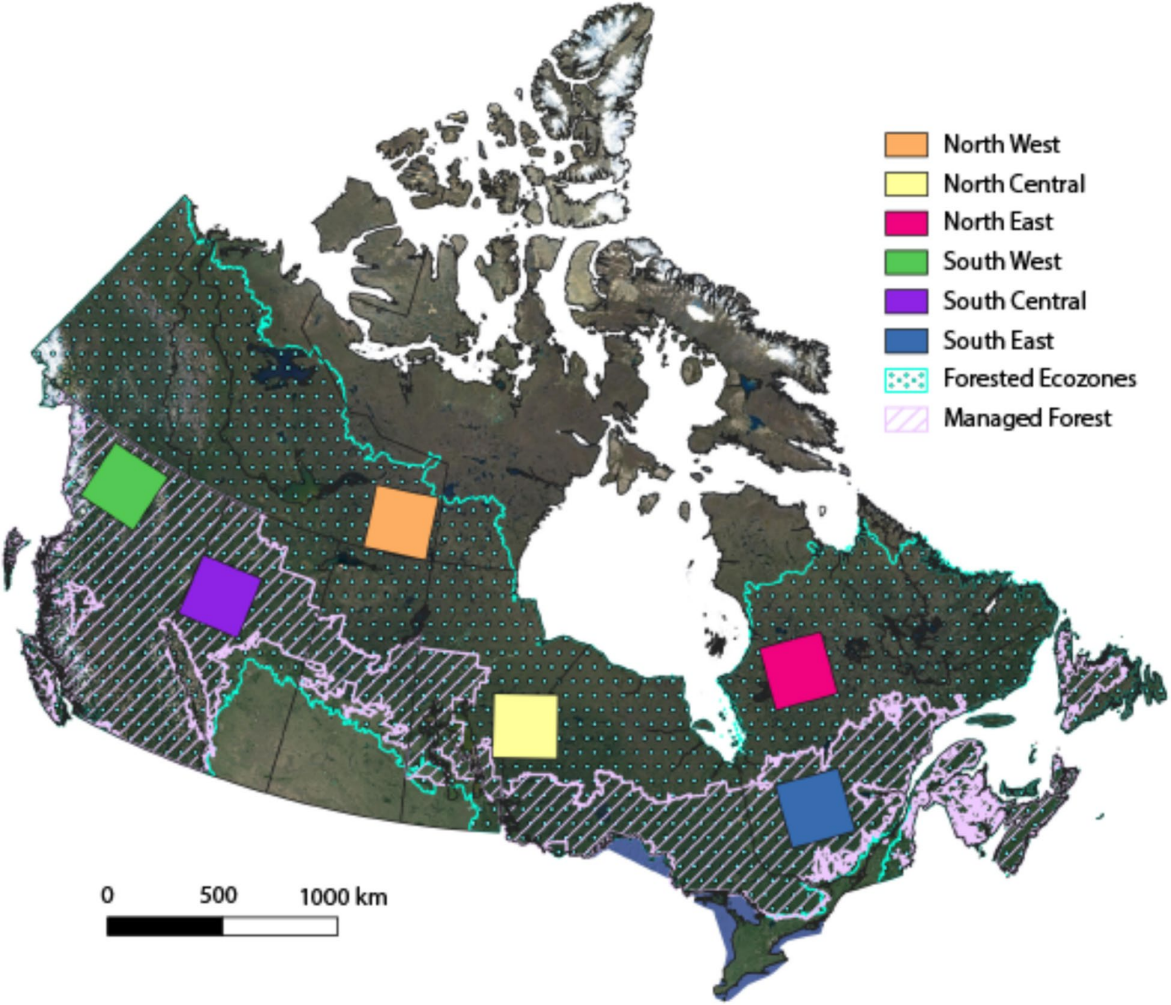
Study area map showing the six study areas along with forested ecozones and area with managed forest. The North West, North Central, and North East are in the unmanaged boreal, while study areas South West, South Central, and South East are in the managed boreal

The boreal forest represents over 75% of Canada’s forests, covering over 280 Mha [15]. Of this, 54% is considered ‘managed’, which includes lands managed for sustainable harvest, lands under protection from natural disturbances (e.g. fire and insect suppression), and protected areas such as parks that are managed to conserve ecological values [50, 81]. Climate in the boreal varies across Canada; from east to west there is a moisture gradient, with areas closer to the coast having higher levels of precipitation than interior areas, while a temperature gradient exists from south to north, leading to closed forests in the south to open forest and forest-tundra in the north [12]. Additionally, ecosystem drivers such as fire, insects, and disease also differ in frequency and intensity across these two gradients [12]. The fire return interval in the boreal forest can vary from 70 to 250 years depending on location, climate, wind speed and direction, and fuel load in forest floor” [49]. Due to this heterogeneity across the boreal, six 300 by 300 km study areas were selected to represent a cross-section of the environments and climates that can be expected in the boreal (Fig. 1, Table 1). These study sites were chosen to capture regional variation in climate and forest composition across the boreal ecozones of Canada as well as being large enough to capture within-region heterogeneity. Three southern sites are in what is considered the managed boreal, while three northern sites are in the unmanaged boreal. Table 1 provides a summary of study area location as well as common species, while Fig. 1 shows their location within Canada.

**Table 1:** Dominant tree species in each study area. Deciduous species % = number of pixels classified as deciduous tree species. Eligible planting area = how much of the area within each focus site was simulated as being planted

Focus Site #	Study area center (lat/long)	Dominant Coniferous Species	Dominant deciduous species	Deciduous Species %	Managed Status	Eligible planting Area (ha)
North West	61.4, -105.27	*Picea mariana*	*Betula papyrifera*	0.4	No	4.68 × 10^5^
North Central	53.46, -93.7	*Picea mariana*	*Populus tremuloides*	8.1	No	1.78 × 10^5^
North East	54.32, -73.95	*Picea mariana*	*N/A*	0	No	1.10 × 10^6^
South West	58.30, -128.09	*Pinus contorta*	*Populus tremuloides*	2.7	Yes	1.34 × 10^6^
South Central	56.09, -117.49	*Picea mariana*	*Populus tremuloides*	53.1	Yes	1.97 × 10^6^
South East	48.63, -75.33	*Picea mariana*	*Betula papyrifera*	26.4	Yes	2.16 × 10^5^

### The 3-PG model

3-PG is a process-based model based on established biophysical relationships used for simulating forest growth at a monthly time step [54]. The model requires species specific physiological characteristics, as well as the following input data: maximum and minimum temperature (°C), precipitation (mm), number of frost days, radiation (MJ m^−2^ d^−1^), and estimates of soil fertility as well as water storage capacity. 3-PG includes the following simplifications: a) that major trends can be adequately captured by monthly mean climate data, b) that the variable which most limits tree growth in a given month is sufficient, c) that net primary production and respiration are approximately equal fractions of gross photosynthesis, d) as leaf area index (LAI) reaches and exceeds 3.0, a constant canopy conductance is reached, and e) the proportion of biomass allocated to roots increases with drought, and decreases with nutrient availability [87].

In the model, the utilized portion of photosynthetically active radiation is determined by a series of modifiers [54], which take a value between 0 (system shut-down) and 1 (no constraint). The modifiers are derived from constraints imposed by the following: stomatal closure associated with high day-time atmospheric vapour pressure deficits (fVPD), soil water balance (fSW), effects of sub-freezing temperatures (fFrost), and a temperature function that regulates photosynthetic capacity seasonally [19, 83]. 3-PG has the capacity to predict stand characteristics such as stem density, mean tree diameter, basal area, standing volume, as well as above- and belowground biomass [18, 22]. 3-PG was originally designed to be used with evergreen species, and as such, litterfall equations were not intended for deciduous species with annual litterfall events [4, 78].

Researchers have overcome this limitation in a number of ways, such as modifications to the litterfall, leaf biomass (Wf), and specific leaf area (SLA) parameters [32]. Litterfall has been modified in a number of ways in different studies, such as by averaging the litterfall amount over the course of the year [30, 37, 72, 91]. Other studies have modified Wf in order to simulate leaves falling off ever year, both Forrester and Tang [31] and Jégo et al. [44] reset the value every spring to simulate the annual loss in biomass. SLA has been modified to change on a monthly timestep (as opposed to annually), in order to simulate leaf maturity changing on an annual basis [44]. Finally, the frost-day modifier can be set to a temperature to nullify growth calculations during winter months, which effectively suspends carbon allocation during these months. For further information regarding model development and calibration, readers are directed to Landsberg & Waring [54], and Landsberg et al. [55]. A comprehensive review of the recent use of the model can be found in Gupta & Sharma [36].

### Sources of data

#### Climate

Future climate data was gathered using the ClimateNA package [60, 85]. This software provides historical and future projections for key climate variables across North America, by downscaling monthly PRISM and ANUSLIN interpolated climate data layers into scale-free point estimates of climate values [25, 43, 85]. Future climate projections were obtained for three Shared Socioeconomic Pathway (SSP) scenarios (SSP1-2.6, SSP2-4.5, and SSP3-7.0) from the 13-GCM ensemble model described in Mahony et al. [60]. SSPs represent different development pathways that align with the IPCC’s Sixth Assessment Report (Masson-Delmotte, V., P. et al. [64]), and take into account a variety of different socio-economic factors [76]; [89]. Monthly normals for three future time periods were downloaded: 2021–2040, 2041–2060, and 2061–2080. Climate scenarios are summarized in Table 2.

**Table 2:** Climate scenarios simulated for different planting scenarios

Scenario name	IPCC SSP	Description	Warming between 2080 and 2100
CS1	SSP1-2.6	Sustainable scenario: low challenges to mitigation and adaptation. Optimistic development, resource efficiency	**1.3 – 2.4 °C**
CS2	SSP2-4.5	Middle of the road scenario: medium challenges to mitigation and adaptation. Current development trends continue	**2.1 – 3.5 °C**
CS3	SSP3-7.0	Pessimistic scenario: challenges to development. Countries focus on achieving energy and food security goals within their own regions at the expense of broader-based development	**2.8– 4.6 °C**

#### Landcover

Medium resolution (30 m) landcover information of Canada’s forested ecosystems was gathered from Canada’s National Terrestrial Ecosystem Monitoring System (NTEMS) project [41, 88]. This information included the following: landcover type which is produced from annual time-series of Landsat image composites [39, 40]. Forest extent, which is based on the Food and Agricultural Organization of the United Nations (FAO) definition of forest [90]. This layer takes into account land use, and trees which are removed by fire and harvested and remain forest. Forest age, which based on Landsat and MODIS imagery [61], and tree species, which is based on 2019 Landsat composites, geographic and climate data, elevation derivatives, and remote sensing derived phenology [39, 40],

#### Soil inputs

Soil fertility was modeled from the Canada-wide global soil carbon dataset, as high soil organic carbon (SOC) leads to increased soil fertility [28]. The soil organic carbon layer was cropped to the extent of the boreal, before being transformed and scaled. High SOC values (> 200 tons/ha) were scaled to a fertility value of 1, while low SOC values (> 40 tons/ha) were scaled to a fertility value of 0.45. Due to missing data from the SOC dataset, areas containing no data were given a value of 0.5. Finally, a smoothing filter was applied in terra [42].

Maximum available soil water (MASW, mm) was derived using the global ASTER Digital Elevation Model (DEM. [82]). A topographic wetness index (TWI) was created using DEM-derived slope and catchment areas using WhiteboxTools [57] following the approach of [23]. TWI was then scaled to MASW using a regression to capture variability across the landscape, and provide consistency for values across all study sites from 100 to 300 mm.

### Simulations

#### Species parameterization

For each study area, we modelled growth for the leading coniferous species, specifically black spruce (*Picea mariana*), and lodgepole pine (*Pinus contorta*). The parameterization of black spruce was based on previous 3-PG parameterisations of similar species, including white spruce [3], Norway spruce (*Picea abies*) [30], and Sitka spruce (*Picea sitchensis*) [69, 86]. Lodgepole pine parameters were based on Coops & Waring [21], and Meyer et al. [67]. We also modeled growth of a ‘generic’ deciduous species, based on the parameterization of hybrid poplar by Headlee et al. [37].

#### Planting scenarios and rules

Areas suitable for planting were identified following the methodology outlined by Drever et al. [26], and applied by Du Toit et al. [27]. In their work, afforestation was eligible to occur in areas that were not previously occupied by forest, i.e. lands which have not been classified as forest in the Landsat record. Additionally, pixels that were unsuitable for forests (specifically water, snow/ice, bryoids, or rock/rubble) were excluded. Pixels eligible for planting therefore had to belong to the following landcover classes: shrubland, and herbs (grasses) [39, 40]. To create different planting scenarios, we used the available soil water layer and normalized it between 0 and 1 (normalized wetness, nW). This layer was stratified into three categories; dry (< 0.33), wet (> 0.67), and medium (0.33–0.67). We then created three planting scenarios, with planting simulation 1 (SM1) representing a baseline of only coniferous planting. In simulation 2 (SM2), we simulated deciduous planting on all eligible pixels. In simulation 3 (SM3), we simulated planting deciduous in the driest areas (< 0.33), and coniferous in the wettest areas (> 0.67), while the medium wetness areas were randomly allocated with either a value of deciduous or coniferous with a 50% chance of either class being assigned. while the medium wetness areas were planted randomly with a 50/50 mixture of coniferous and deciduous species.

**Fig. 2:**
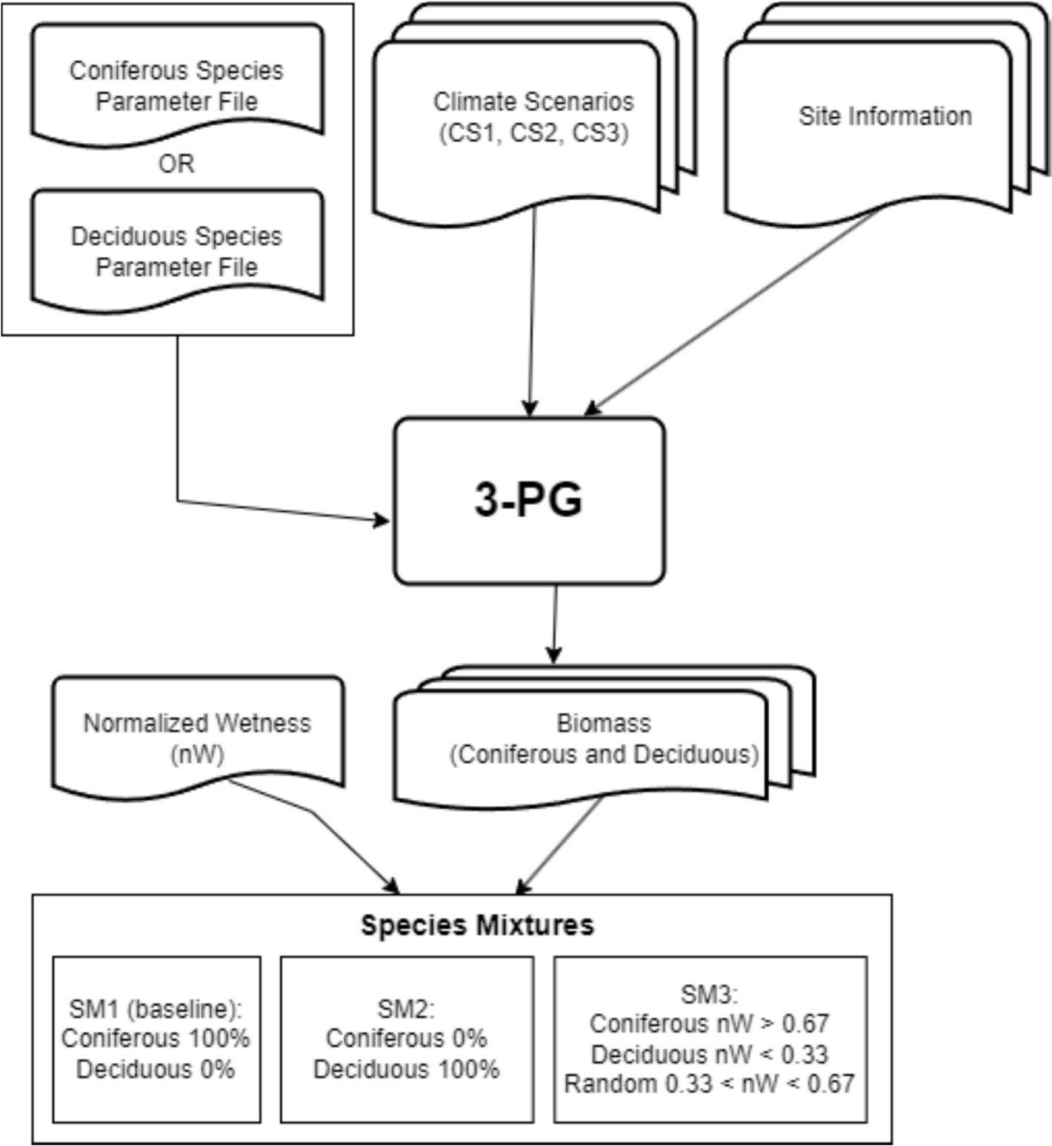
Workflow to simulate species mixtures

Following this, growth could be simulated under the following conditions; a starting stem density of 1650 stems per hectare, and planting in 2025. Simulations were run for the dominant coniferous species in each study area, as well as the deciduous species, from 2025 to 2080 under the three IPCC defined climate scenarios (Table 2). Canopy quantum efficiency was modified for each species to account for the effect of increased atmospheric CO_2_ under each scenario; mean global CO_2_ values were calculated in 20-year periods for each climate scenario based on Meinshausen et al. [65]. Simulations are summarized in Table 3, while the workflow is summarized in Fig. 2.

**Table 3:** Simulations of different planting scenarios under different climates, with 100% coniferous, 100% deciduous, and mixed planting based on nW (normalized wetness)

Planting Scenario	Climate scenario	PlantingrRule
1	SSP1-2.6	Coniferous 100%, Deciduous 0%
2	SSP2-4.5	Coniferous 100%, Deciduous 0%
3	SSP3-7.0	Coniferous 100%, Deciduous 0%
4	SSP1-2.6	Coniferous 0%, Deciduous 100%
5	SSP2-4.5	Coniferous 0%, Deciduous 100%
6	SSP3-7.0	Coniferous 0%, Deciduous 100%
7	SSP1-2.6	Deciduous: nW < 0.33, Coniferous: nW > 0.67, Random: 0.33 < nW < 0.67
8	SSP2-4.5	Deciduous: nW < 0.33, Coniferous: nW > 0.67, Random: 0.33 < nW < 0.67
9	SSP3-7.0	Deciduous: nW < 0.33, Coniferous: nW > 0.67, Random: 0.33 < nW < 0.67

To assess differences in planting simulations, we employed the Kruskal–Wallis test (alpha = 0.05) in two ways. First, we compared planting simulations within each climate scenario to identify significant differences (i.e. planting simulations were compared to one another in CS1, CS2, and CS3). Secondly, we compared simulations across different climate scenarios to explore the overall effect of climate on planting outcomes (i.e. each simulation was compared to itself across CS1, CS2, and CS3). Following these Kruskal–Wallis tests, we conducted Dunn’s post-hoc tests for pairwise comparisons between each planting simulation within each climate scenario, and again across all climate scenarios. This allowed us to identify which specific simulations were statistically significantly different from one another in the final year of the simulation (2080).

#### Effects of water stress

Finally, to understand the potential effects of seasonal water stress on stand growth, the soil water modifier (fSW) and vapor pressure deficit modifier (fVPD) were modelled. The fSW modifier indicates water stress by accounting for the difference between total monthly transpiration (mm), calculated using the Penman–Monteith equation, and monthly precipitation [20]. This modifier is impacted by the available soil water for a given month. The fVPD modifier reflects the correlation between the vapor pressure deficit and leaf stomata,when vapor pressure deficit is low, stomata close, leading to decreased growth as CO_2_ cannot be absorbed by leaves [53, 54].

These modifiers were modelled for every month of the stands at age 55 (2079) over each study area for both deciduous and coniferous planting. To compare the effect of species on each modifier, the monthly modifiers were then differenced across the landscape with changes in the modifiers greater than 5% flagged for further investigation.

## Results

### Planting scenarios

**Fig. 3:**
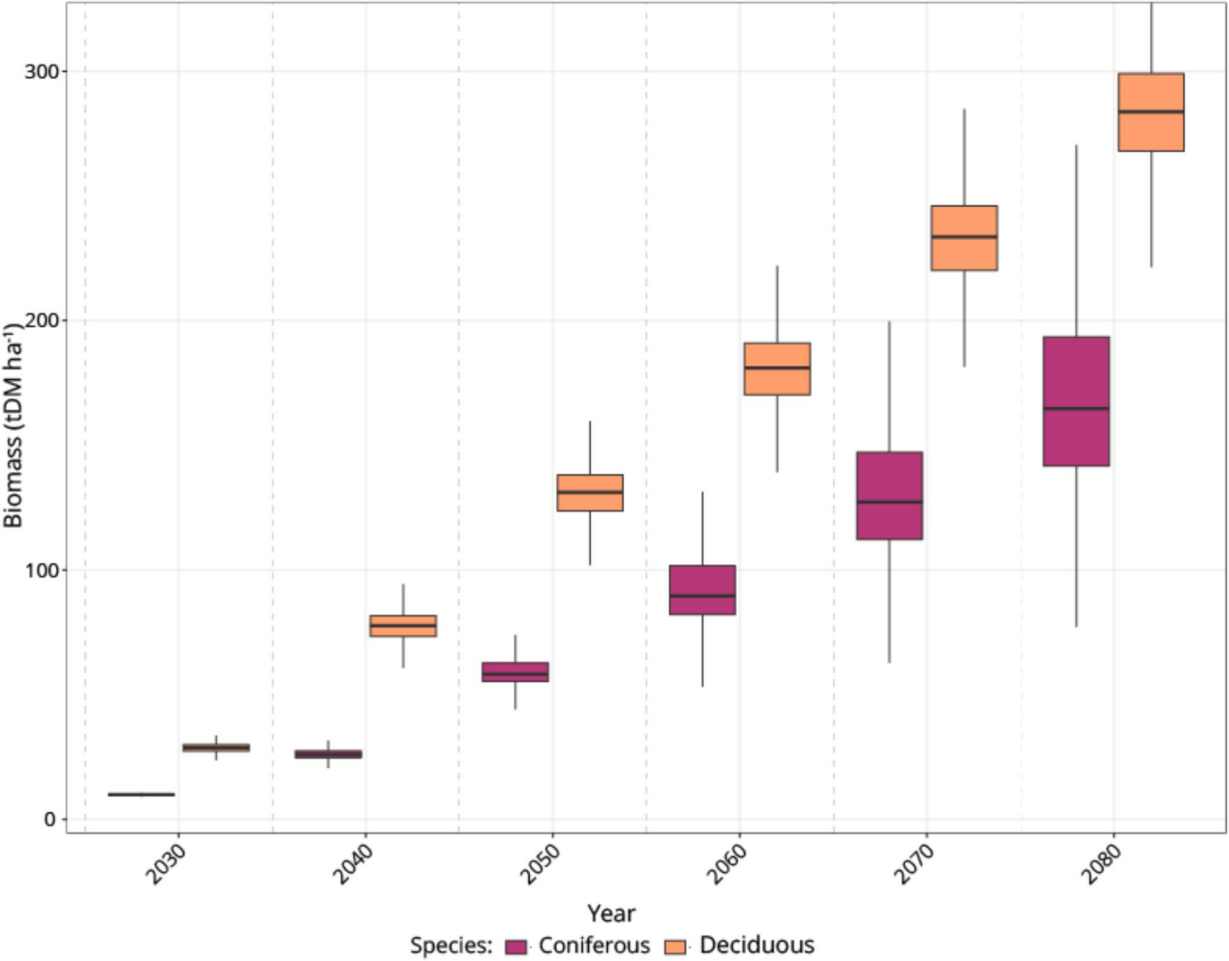
Comparison of biomass (tDM ha.^−1^) accumulation over time for coniferous (purple) and deciduous (orange) species. The box plots show the distribution of biomass in the South West Study area, every 10 years, from 2030 to 2080 under Climate Scenario 2

**Fig. 4:**
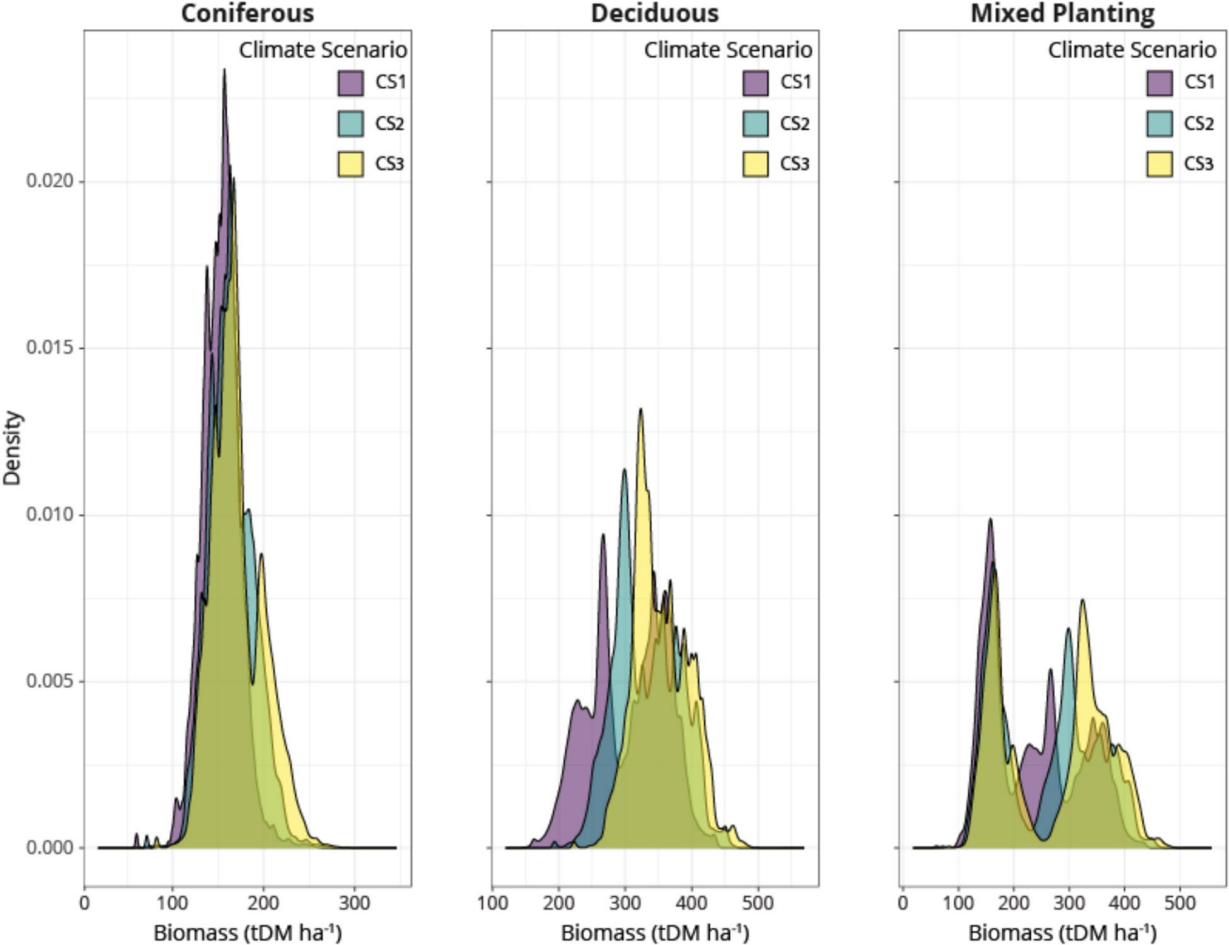
Density plot of biomass (tDM ha.^−1^) distributions by planting type under different climate scenarios (CS1, CS2, and CS3). The density illustrates the frequency distribution of land area across a continuous range of biomass values for the final simulated year (2080)

Across the six study areas, 5,270,958 ha of land met the conditions for planting (i.e. never forested and appropriate landcover type), compared to approximately 31,385,237 ha of existing forest land in total. Table 4 provides a summary of the biomass accumulated across climate scenarios for different planting simulations. Planting simulation 2 (SM2) had the highest biomass accumulation by 2080 across all three climate scenarios, with a mean of 351.7 (tDM ha^−1^) under climate scenario 3 (CS3). The coniferous planting simulation (SM1) was consistently lowest, accumulating 171.1 (tDM ha^−1^) under the same planting scenario. Figure 3 shows the accumulation of biomass over time under climate scenario 2 (CS2), with biomass for every decade from 2030 to 2080. The figure shows that biomass in the deciduous simulation is consistently higher than coniferous, which can also be seen in the horizontal distribution of biomass values in Fig. 4.

**Table 4:** Summary of the mean, median, and standard deviation (SD) of biomass (tDM ha^−1^) across all study sites, accumulated by 2080 across different climate scenarios (CS1, CS2, CS3), and different planting scenarios (SM1, SM2, SM3)

Climate Scenario	Conifer (SM1)			Deciduous (SM2)			Mixed (SM3)		
	Mean (tDM ha^−1^)	Median (tDM ha^−1^)	SD (tDM ha^−1^)	Mean (tDM ha^−1^)	Median (tDM ha^−1^)	SD (tDM ha^−1^)	Mean (tDM ha^−1^)	Median (tDM ha^−1^)	SD (tDM ha^−1^)
CS1	153.4	154.1	20.7	302.4	310.7	60.7	234.0	224.0	84.8
CS2	164.1	162.4	24.8	330.6	327.5	52.8	255.6	272.4	90.8
CS3	171.1	167.0	28.2	351.7	347.1	47.3	271.6	307.7	96.8

The distribution of biomass accumulation across different climate scenarios can be seen in Fig. 4. In general, the distribution of the coniferous planting under each climate scenario (left) contains one strong peak, although a second peak appears in CS3. Deciduous distributions remain relatively consistent regardless of climate scenario, although a clear shift towards higher biomass accumulation occurs in CS3. Under the mixed planting simulation (SM3), we can see a multimodal distribution under CS1, while under CS3 this distribution tends towards a bimodal distribution, with distinct peaks reflecting the mixed planting of coniferous and deciduous species. Comparing distributions within climate scenarios (right) each planting simulation has a distinct distribution.

The results of the Kruskal–Wallis test for both planting simulation by climate scenario, and climate scenario by planting simulation produced statistically different results (p < 0.05). The post-hoc Dunn’s test for pairwise comparisons of each climate scenario (CS1, CS2, CS3) under different planting simulations (SM1, SM2, SM3) were statistically significant (P.adj < 0.05, Supplementary Table 1), while the same was true for planting simulations (SM1, SM2, SM3) by climate scenario (CS1, CS2, CS3, P.adj < 0.05, Supplementary Table 2).

**Fig. 5:**
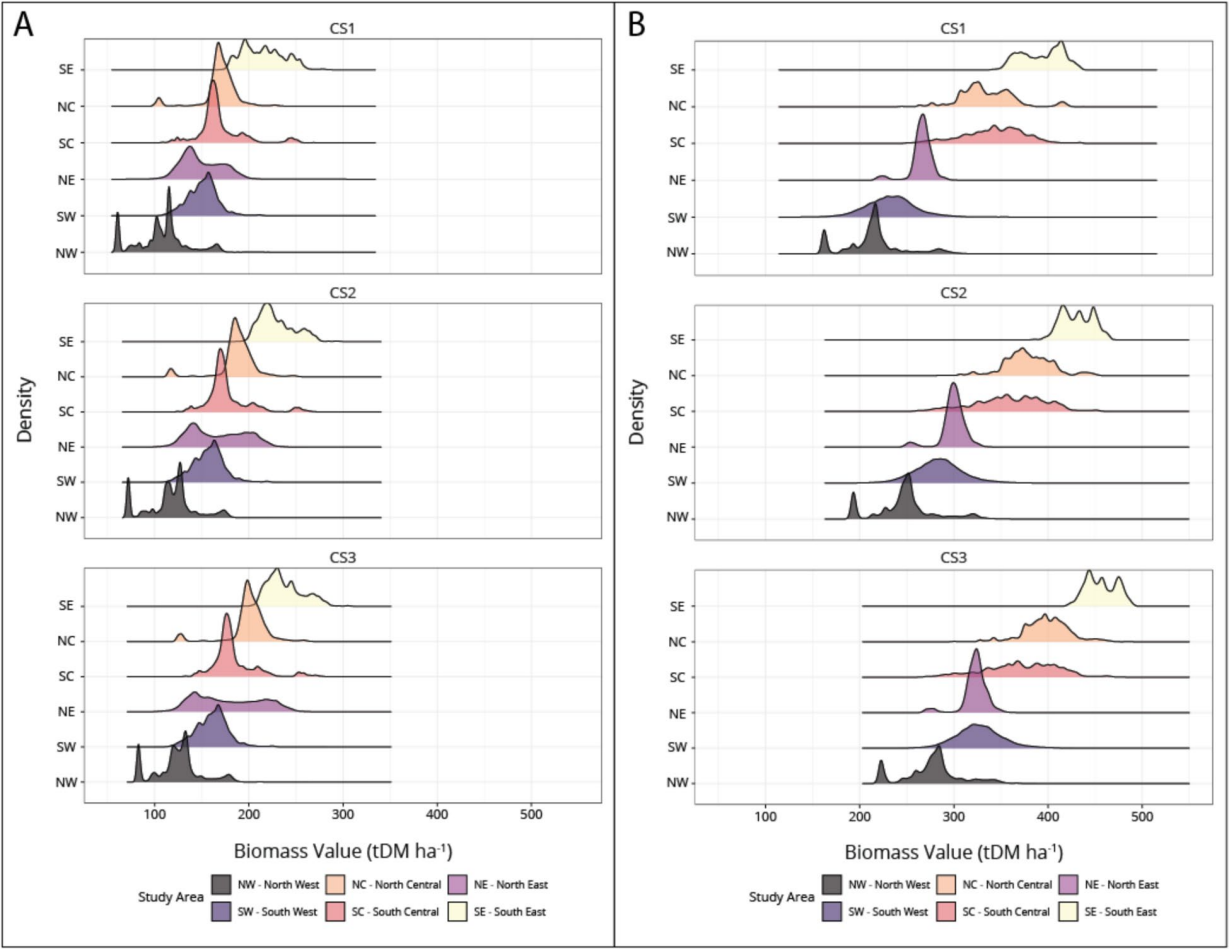
Comparison of growth in different study areas for coniferous (**A**) and deciduous (**B**) species. The density illustrates the frequency distribution of land area across a continuous range of biomass values for the final simulated year (2080), separated by study area

Figure 5 shows growth by study area; with clear variations in growth between study areas. Figure 5A shows simulated growth of conifers, while 5B shows simulated growth of deciduous species. In both cases, growth patterns are largely the same across study areas, with the lowest growth in the northwest study area, and the highest growth in the southeast study area.

### Effects of water stress

**Fig. 6:**
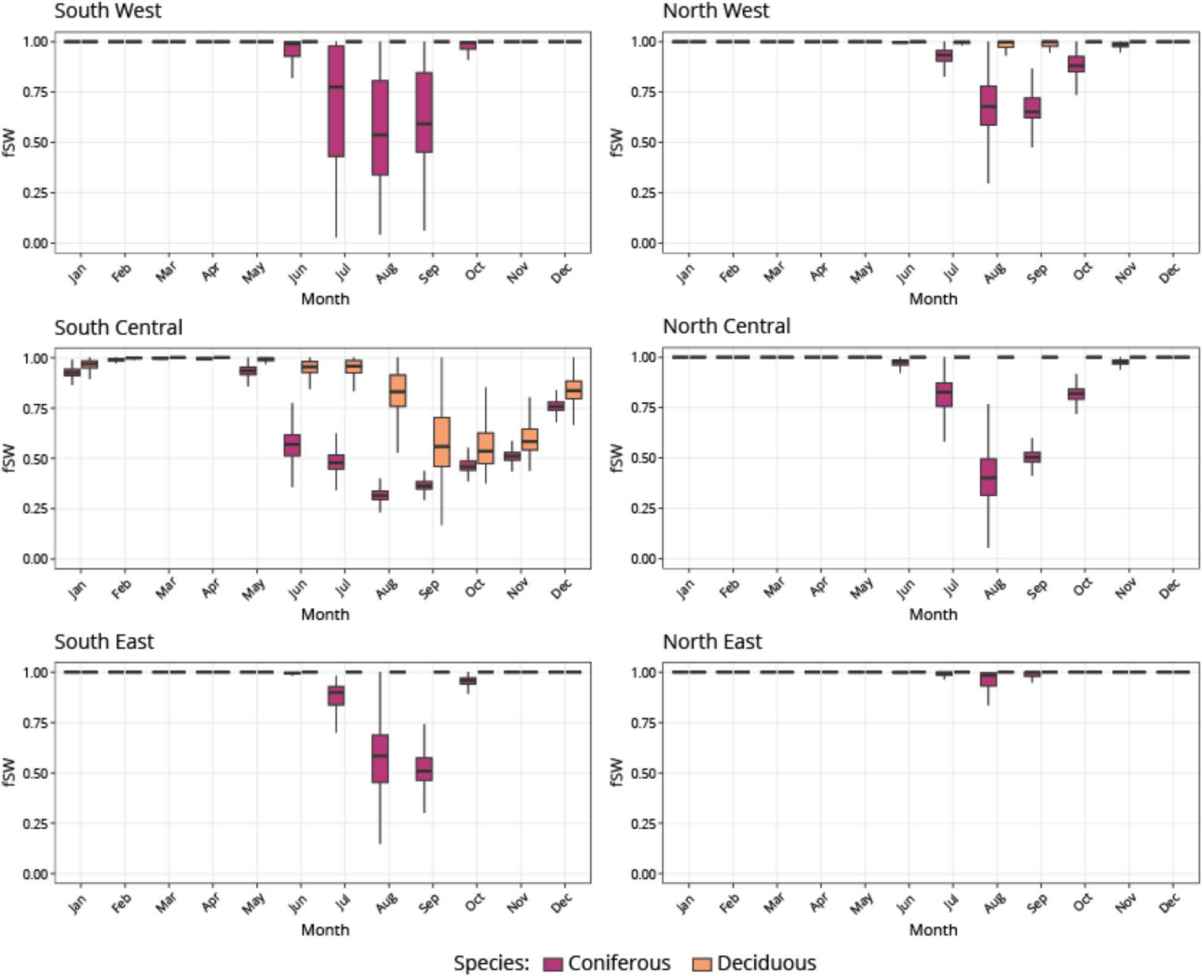
Comparisons of the soil water dependent modifier (fSW) between coniferous and deciduous species for all study areas for every month in 2079 under climate scenario 3 (CS3)

With respect to changes in water use we found no significant differences in the vapor pressure deficit use across the simulations, however soil water stress (fSW) for July, August, and September was identified as being different (> 0.05) in the final year of the simulation for every study area. Figure 6 shows how fSW varies monthly for both deciduous and coniferous species across the year in the year 2079 under CS3 (the most extreme climate scenario). Generally, all coniferous simulations saw increases in water stress in the summer months. In contrast, deciduous saw little water stress through the year for all scenarios, except for the South Central study area. Overall, stress was always greater for coniferous species compared to deciduous species. The water stress in July was on average 25.6% greater for coniferous species compared to deciduous, 65.6% greater in August, and 49.4% greater in September. Northern sites were generally less stressed than southern sites; coniferous simulations were 23.4%, 28.6%, and 29.6% more stressed at the southern (managed) sites in the months of July, August, and September. In contrast, October showed the greatest difference in stress for the coniferous simulations, with the southern sites being 13.9% more stressed on average.

## Discussion

### Results

Our results suggest that the total above ground biomass under the SM2 (deciduous planting) scenario produced the highest level of biomass across all management simulations and as expected biomass was highest under CS3; the warmest climate scenario. This suggests that deciduous species are capable of sequestering large amounts of carbon, as the climate gets warmer. While biomass accumulation increased under all species mixes, the coniferous simulation (SM1) showed the lowest overall increase across the climate scenarios. Biomass accumulation between CS1 and CS3 under the mixed planting simulation (SM3) was close to the midpoint between SM1 and SM2, indicating that the planting mixture was relatively even across the study sites, although this could change based on the proportions chosen for a given planting mixture. The results of the simulation suggest that the impact of planting varying proportions of conifer, deciduous, and mixed species on aboveground biomass differs significantly across climate scenarios. This demonstrates that optimal species composition across the landscape is climate-scenario dependent, and would need to be optimised depending on the local climatic conditions and the future anticipated conditions at each site.

The spatial distributions of biomass across managed and unmanaged boreal stands, as well as study areas, indicate that there is no single optimal planting scenario (Fig. 5), as large variations in biomass accumulation exist across the boreal. Simulations at finer spatial scales would therefore be beneficial to maximizing both forest resilience and biomass accumulation. These localized simulations are therefore beneficial in a number of ways. First, they provide insights into the potential benefits of planting deciduous versus conifers stands with respect to their overall biomass accumulation, secondly it allows other forest ecosystem goods and services, for example habitat for wildlife, to be considered when deciding the optimum species mix and planting scenarios. Critically, however, as these results show the accumulation of biomass over these areas is highly variable. As a result, these simulations can be used to guide where within these larger areas planting attention should be focused thereby ensuring that not only the likely of success of establishment is optimized but also where carbon storage is maximized in order to gain these climate benefits.

### Deciduous afforestation in the face of climate change

The high level of biomass accumulation between 2025 and 2080 in the deciduous planting simulation (SM2) suggests an important role for deciduous planting in future forest management. However, we recognise that while our simulations use future climate predictions, these predictions do not include multiple years of extreme temperatures or precipitation. As a result, we are not able to simulate periods of extended drought which in turn might lead to mortality as well as landscape impacts such as fire. Increasing the mixture of species under these extreme scenarios could generate heterogeneity in forest structure which may in turn reduce vulnerability to large, catastrophic disturbances [33]. For example, fire-adapted conifers may have an advantage in colonizing areas where fires are not frequent enough to prevent trees from reaching cone-bearing age [73], while in areas with short return periods of stand-replacing fires (preventing sexual maturation of conifers), deciduous species may dominate [74]. Additionally, species that produce light seeds designed for wind dispersal, such as poplars and birches might have an advantage for colonizing new areas after a disturbance [73]. Given the goal of natural climate solutions are to mitigate the effects of climate change by increasing carbon sequestration, the planting of a mixture of species during afforestation is a step towards future proofing these approaches, as there is a significant economic component. While we randomly selected cells for planting in a number of our scenarios, in particular in response to soil moisture, to increase this spatial heterogeneity across the study areas future studies can use this approach to target planting specific species that meet other appropriate environmental conditions (i.e. use multiple layers to stratify planting rules) in order to maximize sequestration.

While this study focused on afforestation using different species mixes, we can also draw parallels to reforestation after a stand replacing disturbance. While fire intensity and frequency are expected to increase generally, models suggest that moister parts of the boreal forest will tend to stay moist, whereas drier parts of the range are likely to become drier [75]. As such, it is important for forest managers to select prescriptions that promote forest regeneration and also consider the long-term effects of changing climate and disturbance regimes in a given area [6]. Given that an increase of fire severity in addition to climate warming and drying may lead to an increased ‘deciduousness’ in forest composition [2], the choice to plant these species moving forward makes sense. The benefits of an increase in deciduous species include an increase in surface albedo, as well as higher levels of atmospheric water vapor [2, 5, 8]. These benefits, alongside higher leaf moisture and lower flammability [16, 45] could have a significant effect to counter wildfire disturbance trends.

It is important to contextualize the "afforestation" scenarios modeled in this study with practical forest management strategies in the managed and unmanaged boreal. While our model simulates planting, in reality, forest managers may prioritize natural regeneration of deciduous species (i.e. they will no longer be controlled via juvenile thinning or herbicide to favour conifers), particularly in the managed areas of the boreal. This approach aligns with a dynamic view of conservation, acknowledging that natural processes and disturbances are important for ecosystem resilience [48]. This approach also contributes to diversifying species composition, enhancing overall forest resilience to changing climate and disturbance regimes [71].

### Soil water and vapor pressure deficit modifiers

While no large differences were observed in the vapor pressure deficit modifier (fVPD) between coniferous and deciduous simulations, soil water (fSW) varied considerably over the range of the boreal forest in Canada. Southern sites showed more stress than the northern sites, likely due to increased summer temperatures in the south, as well as longer periods of drought compared to the North. Generally, the simulations suggest that deciduous species are less stressed, and when stress occurs it is less severe and for shorter periods of time than conifer stands.

Planting deciduous species has been shown to effective to counteract drought-related disturbance events; for example, Breil et al. [13] showed that an increase in the deciduous forest fraction reduces the heat intensity during heat periods in most regions of Europe, in part because of deciduous species’ ability to effectively reduce water stress. As such, areas that will suffer from reduced precipitation or decreases in soil moisture due to changes in future climate conditions can benefit from planting deciduous species. Additionally, the modelling of future soil moisture regimes, as well as plant-soil water interactions could be used to inform species selection for afforestation and reforestation [59, 66].

While our research used a generic deciduous parameterization as deciduous simulations are rare in 3-PG, future work should focus on parameterizing additional deciduous species that exist in the boreal, as sensitivity to soil moisture deficits can vary by species [75]. For example, trembling aspen (*Populus tremuloides*), is tolerant of various stresses, including periodic drought and insect defoliation, a factor contributing to its wide distribution one of the most abundant deciduous species in Canada’s boreal forest [11, 49, 56]. Additionally, its extensive clonal root system allows trees to re-sprout after disturbance or stress events [56]. Aspen exhibits conservative water use during high vapor pressure deficits, with stomatal conductance decreasing with soil drying to maintain water potential above critical levels [56]. However, despite these adaptations, severe regional droughts have led to massive mortality and widespread dieback of aspen across large areas of west-central Canadian boreal and parkland forests, prompting concerns for the future in a changing climate [56, 68]. Defoliation events, often interacting with drier periods, have been shown to significantly reduce root starch reserves in mature aspen, increasing the risk of carbon limitation. This reduction can lead to root loss and a cascade of negative effects on water and nutrient uptake which in turn will limit carbon assimilation [51]. This highlights the need for accurate parametrization of boreal deciduous species, capturing their complex and sometimes counter-intuitive responses to climate change, especially when interacting with other environmental stressors.

### Limitations, challenges and future work

#### Species parameterization

The coniferous species which form the basis of this research (black spruce and lodgepole pine) were parameterized for 3-PG based on Du Toit et al. [27] which included an accuracy assessment. The generic deciduous species represented in 3-PG is based on the parameterization of hybrid poplar by Headlee et al. [37] due to the absence of a species specific parameterization of a deciduous boreal species. The use of hybrid poplar as a proxy for trembling aspen could bias productivity estimates upward, potentially leading to an overestimation of biomass accumulation in the deciduous scenarios. This limitation of requiring species specific parameters in 3-PG is common in some regions as many boreal species lack comprehensive parameter sets and proxies from physiologically similar species are often used (e.g., [18, 22]). Nevertheless, the choice to plant a hybrid poplar may be relevant when assessing the goals of deciduous afforestation as a nature-based adaptation solution; a fast growing species which thrives under future climate conditions may be preferential to marginal deciduous species currently found in the boreal. While the focus of this study is on relative differences between climate scenarios rather than absolute productivity, the uncertainty introduced by species matching should be considered when interpreting the results. Future work should prioritize the development and integration of species-specific parameterizations for key boreal species to reduce model uncertainty and improve projections of forest carbon dynamics under climate change.

Our focus in this study has been on above ground biomass and carbon since these are the variables that are most often used and reported on, when examining nature-based climate solutions. A key benefit of 3-PG, however, is the capacity for it to also predict overall stand volume as well as merchantable volume, which are variables much more relevant to forest production and timber extraction. As a result, it would be a simple extension of this work to examine these benefits of these afforestation efforts with respect to these variables rather than solely biomass and carbon. It is important to recognize however that our focus is the northern unmanaged boreal forests where currently timber is not harvested commercially.

Whilst changes in above ground biomass and carbon are evident under a changing climate this additional biomass is likely insufficient to support commercial timber extraction especially given then associated infrastructure which would be required in order to support these types of industrial activities. As a result, while examining these results with respect to standing volume is possible it is likely less relevant given the remoteness of many of these sites for the timber industry.

#### Soil characterization

Soil characterization in 3-PG depends on fertility, available soil water, and predominant soil characteristic (sand to clay). These parameters can be very difficult to find at a high spatial resolution, particularly in remote areas. In this research we used proxies such as soil carbon for fertility, and the use of a digital elevation model for available soil water, however these parameters are still dependent on the resolution and accuracy of the original datasets. To further examine the effects of the soil water modifier in the boreal, future research should investigate other resources to ensure accuracy of soil characteristics, as this would provide the potential to model plant-soil interactions and effects using 3-PG. For example, [66] investigated the 2015 and 2018 droughts in Switzerland; similar analyses could incorporate 3-PG provided soil data quality was high. 

#### Effects of fire and albedo in the boreal

This research focused on projecting forest growth and physiological responses to environmental factors for various afforestation scenarios, and does not explicitly simulate the effects of fire and albedo. The 3-PG model predicts stand growth and not landscape processes such as fire, and as a result we do not model changes in species composition in response to stress or fire, or the dynamic feedback associated with successional pathways under changed species regimes. However, existing literature demonstrates that a greater proportion of deciduous species can influence fire regimes by reducing fuel loads, increasing leaf moisture, and lowering flammability. For example, Hély et al. [38] showed that fires were less intense and spread more slowly in deciduous stands. Additionally, Bergeron et al. [9] highlighted how strong deciduous components in mixedwood forests lead to smaller, lower-intensity fires, while Marchal et al. [62] demonstrated that converting conifer-dominated landscapes to hardwoods can substantially decrease fire size and frequency. Furthermore, Mulverhill et al. [70] found that forest type was an important variable for predicting burn probability, due to fires preferentially burning coniferous rather than deciduous trees. It is crucial to integrate wildfire modeling into future work to account for the effects of on forest regeneration, as well as impacts on carbon to provide a more complete NCS assessment.

Similarly, we did not account for changes in surface albedo associated with shifts in forest composition. It has been shown that an increase in surface albedo of deciduous broad-leaved forests compared to evergreen forests could result in year-round cooling [2, 7, 8, 14]. However, while this albedo-driven feedback can be significant it is not represented in the 3-PG model framework. As a result, our analysis does not address the full range of climate impacts associated with afforestation or species shifts, highlighting the need for studies that integrate both carbon and biophysical processes. Additionally, while albedo changes may increase from deciduous to broad-leaved forests, we did not account for the potential decrease in albedo that can occur when changing the landcover from grasses to trees, which could potentially exacerbate global warming due to changes in albedo [29]. Future studies should consider both fire and albedo because these factors can have a substantial effect on the carbon balance in the boreal. Quantifying these relationships and integrating them into future modelling efforts can help researchers provide more comprehensive assessments of ecosystem processes.

#### Time Horizon of carbon sequestration

The timescale of carbon sequestration is an important aspect when considering afforestation and other natural climate solutions. While deciduous trees may sequester more carbon in the short and medium term, it is difficult to predict the above ground biomass in these forests beyond the 100–200 years time. If deciduous forests are generally not as long lived as coniferous forests, the implications of their role in the carbon cycle must be considered. Additionally, if looking for natural climate solutions to perform multiple roles (e.g. enhance biodiversity), it is important to start considering the linkages between carbon sequestration, ecosystem biodiversity, resilience, and restoration (e.g. [24]). Process-based models (e.g. 3-PG, PnET, 3D-CMCC FEM) can play a major role in approaching these holistic climate solutions due to their ability to model different species across the landscape in a spatially explicit manner [1, 17, 54].

#### Carbon accounting of soil and dead organic Matter

While the primary focus of this study was on aboveground biomass (AGB) accumulation, other carbon pools could be investigated in future study, as 3-PG models can predict biomass in other pools such as roots, soil organic carbon (SOC) and dead organic matter (DOM) was outside the scope of this study. These carbon pools can be relatively large; for example Michaelian et al. [68] highlighted that DOM can have a marked effect on carbon emissions following decomposition, while Robinson et al. [77] emphasise the importance of incorporating pools such as SOC for completeness of carbon removal estimates. Future research should integrate these carbon pools, potentially using landscape models with extensions for carbon succession, such as LANDIS-II with the ForCS extension [52], to provide a more integrated understanding of the climate change mitigation potential.

## Conclusion

This research investigated the impact of deciduous vs. conifer afforestation on biomass accumulation in the Canadian boreal under a changing climate Using the 3-PG model, we simulated the planting of deciduous, coniferous and mixed forests in the Canadian boreal. Forests were grown under three climate scenarios to 2080; we found that increased biomass accumulation in deciduous forests stands across all sites, and that the more biomass was accumulated under the warmest climate scenario (CS3) than others. Additionally, we found that coniferous species were typically more stressed in the summer months both with respect to the severity of the stress and its duration than deciduous species and this degree of stressed increased as stands aged, under an increasingly warm climate to 2100. Process-based models can provide critical insights for informing adaptive forest management, particularly under a changing climate. Future work should prioritize multi-model ensembles to enhance robustness when studying long-term forest carbon dynamics. This study highlights the importance of modelling and consideration of different planting scenarios in decision-making to ensure successful resource allocation. Deciduous afforestation in the boreal can serve as a crucial nature-based adaptation solution, enhancing forest resilience and providing significant co-benefits such as wildfire risk reduction and in addition to carbon sequestration.

## Supplementary Information

Supplementary Materials 1

## Data Availability

The 3-PG model is available and is free and open to use. Code is available here: —https://github.com/IRSS-UBC/3pg Data utilised in this study is also free and open and available at the following locations: — Canada’s National Terrestrial EcosystemMonitoring System (NTEMS) landcover information: https://opendata.nfis.org/mapserver/nfis-change\_eng.html —Climate projections for North America: https://climatena.ca/spatialData](https:/climatena.ca/spatialData) Species which have been parameterised for 3-PG are listed here: —https://3pg.forestry.ubc.ca/software/](https:/3pg.forestry.ubc.ca/software) Species parameterisations used in the study are available from the authors. Access to the spatial layers generated by this research are available upon request pending approval by all authors.
