# Supplementary material for: Deciduous afforestation as a natural climate solution: impacts on biomass and carbon sequestration in boreal forests of Canada
Source: Carbon Balance Manag. 2026 Jan 3;21:22. doi: 10.1186/s13021-025-00385-2 (PMC12836844; doi:10.1186/s13021-025-00385-2)
Supplement: Supplementary file 1 — Supplementary Materials 1 [file 13021_2025_385_MOESM1_ESM.docx]

## Supplementary Material

Supplementary Table 1: Dunn’s Test for pairwise comparisons of climate scenario (CS1, CS2, CS3) under different planting mixes (SM1, SM2, SM3). Z = Z-score, P.adj = adjusted p-value.

| Comparison | Coniferous (SM1) | | Deciduous (SM2) | | Mixed (SM3) | |
| --- | --- | --- | --- | --- | --- | --- |
|  | Z | P.adj | Z | P.adj | Z | P.adj |
| CS1 - CS2 | -172.7 | 0.0 | -170.9 | 0.0 | -95.6 | 0.0 |
| CS1 - CS3 | -261.1 | 0.0 | -334.7 | 0.0 | -171.5 | 0.0 |
| CS2 - CS3 | -88.7 | 0.0 | -164.1 | 0.0 | -76.1 | 0.0 |

Supplementary Table 2: Dunn’s test for planting mix (SM1, SM2, SM3) by climate scenario (CS1, CS2, CS3). Z = Z-score, P.adj = adjusted p-value.

| Comparison | CS1 | | CS2 | | CS3 | |
| --- | --- | --- | --- | --- | --- | --- |
|  | Z | P.adj | Z | P.adj | Z | P.adj |
| Conif (SM1) – Decid (SM2) | -404.832 | 0 | -403.196 | 0 | -402.782 | 0 |
| Conif (SM1) – Mix (SM3) | -221.923 | 0 | -220.204 | 0 | -221.971 | 0 |
| Decid (SM2) – Mix (SM3) | 182.448 | 0 | 183.4059 | 0 | 180.601 | 0 |
